# Excitonic effects in the optical spectra of Li_2_SiO_3_ compound

**DOI:** 10.1038/s41598-021-87269-w

**Published:** 2021-04-08

**Authors:** Nguyen Thi Han, Vo Khuong Dien, Ming-Fa Lin

**Affiliations:** 1 Department of Physics, National Cheng Kung University, 70101 Tainan, Taiwan; 2 Department of Chemistry, Thai Nguyen University of Education, 20 Luong Ngoc Quyen, Quang Trung, Thai Nguyen City, Thai Nguyen Province Vietnam; 3 Hierarchical Green-Energy Materials (Hi-GEM) Research Center, National Cheng Kung University, Tainan, Taiwan

**Keywords:** Applied physics, Condensed-matter physics, Optical physics

## Abstract

Li_2_SiO_3_ compound exhibits unique electronic and optical properties. The state-of-the-art analyses, which based on first-principle calculations, have successfully confirmed the concise physical/chemical picture and the orbital bonding in Li–O and Si–O bonds. Especially, the unusual optical response behavior includes a large red shift of the onset frequency due to the extremely strong excitonic effect, the polarization of optical properties along three-directions, various optical excitations structures and the most prominent plasmon mode in terms of the dielectric functions, energy loss functions, absorption coefficients and reflectance spectra. The close connections of electronic and optical properties can identify a specific orbital hybridization for each distinct excitation channel. The presented theoretical framework will be fully comprehending the diverse phenomena and widen the potential application of other emerging materials.

## Introduction

Li_2_SiO_3_, which belongs to alkali silicate compounds, has received a great amount of scholarly interest mainly owing to its wide range possible applications in various fields^1–5^. It can be core components in the lithium-based batteries (LIBs)^6–14^ governing modern electronic devices, can be used in electro-optic applications or be utilized as CO_2_ sorbent to fight global warming^15–17^. Indeed, the amorphous thin film Li_2_SiO_3_ exhibits a reliable ion conductivity of 2.5 × 10^–8^ S.cm^−1^ at the room temperature^12^, the coating of Li_2_SiO_3_ layers also help to improve the electrochemical performance of the electrode, as for low cost and safely batteries^18–20^, the Li_2_SiO_3_ compound expresses as a strong candidate for the electrolyte/electrode materials in LIBs^10,21–24^. For greenhouse gas collection, CO_2_ molecules absorption on the surface of the Li_2_SiO_3_ compound is separated into non-toxic and useful substances through reaction: Li_2_SiO_3_ + CO_2_ ↔ SiO_2_ + Li_2_CO_3_^16^. On the other hand, many previous studies also showed that the Li_2_SiO_3_ compound has been actively proposed as a luminescent material^25,26^. The outstanding optical properties, the polar symmetry of the orthorhombic structure suggest that this material is suitable for piezoelectric, pyroelectric, and electro-optic applications^27–30^.

Despite the potential applications of this material have been identified, the fundamental properties as electronic and optical properties are under debates.

Up till now, orthorhombic Li_2_SiO_3_ compound has been successfully synthesized by various methods^31,32^. Its essential physical/chemical properties, such as optimized geometry structures and surface monologies, are usually examined by X-ray diffraction (XRD)^22^, scanning electronic microscopy (SEM)^33,34^ and tunneling electronic microscope (TEM)^22,35^. The energy spectrum depending on frequencies and wave vectors at the valence states, the van Hove singularities in the density of states and the band gap values have not been achieved because the lacking of angle-resolved photoemission spectroscopy (ARPES)^36^, scanning tunneling spectroscopy (STS) and electric conductivity measurements^37^, respectively. Regarding the optical responses, photoluminescence, absorption, and transmission measurements were performed. However, previous studies investigated for doped Li_2_SiO_3_ or measured at high temperature, in which optical properties are strongly affected by surface defects^38–42^.

On the theoretical side, the first principle calculations are frequently utilized to understand the geometric, electronic and optical properties of emerging materials^43–49^. However, this approach will significantly underestimate the band gap values, especially for a large gap semiconductor or insulator materials^50,51^. For instance, the fundamental bandgap is calculated by the density functional theory (DFT) using the Perdew–Burke–Ernzerhof (PBE) parametrization of the generalized gradient approximation (GGA) indicated an indirect band gap of 5.1 eV^52^ for Li_2_SiO_3_ compound. After applying the Heyd-Scuseria-Ernzerhof (HSE) hybrid functional, the band gap value increase to 7.8 eV^25^. Besides, the optical properties of solid materials are strongly affected by the electron–hole interactions. However, to the best of our knowledge, such impacts have been not included in the previous calculations. Most importantly, the significant orbital hybridizations that survive in chemical bonds are rather complicated and responsible for the fundamental properties. Meanwhile, investigations to identify such critical bonding mechanism in this material are still rather limited. Furthermore, the systematic connection of the orbital hybridizations with the electronic properties and excitonic effects in optical properties has not been achieved so far.

The developed theoretical framework, which is established on the significant orbital hybridizations in chemical bonding, is utilized to examine the crucial features in emerging materials. This strategy indicates the optimized geometric structure with position-dependent chemical bonding, the atom dominated energy spectrum at various energy ranges, the spatial charge density distribution due to different orbitals, and the atom- and orbital-projected density of states associated with the overlap of orbitals. Furthermore, the specific orbital hybridizations will be used to interpret the onset of the optical frequency, stable excitonic states, a lot of prominent absorption structures, so strong Plasmon mode in terms of the dielectric functions, energy loss functions, absorption coefficients, and reflectance spectra under the distinct electric polarization. The predictions in this letter require highly resolved experimental measurements.

## Computational details

In this paper, we used the first principle calculations method via the Vienna Ab-initio Simulation Package (VASP)^53^ to perform the optimization structure and calculate the electronic and optical properties. The Perdew-Burke-Ernzerhof (PBE) generalized gradient approximation was used for the exchange–correlation functional^54^. The projector augmented wave (PAW) pseudopotential was utilized to treat core electrons^55^. The cutoff energy for the expansion of the plane wave basis was set to 500 eV. The Brillouin zone was integrated with a special k-point mesh of 25 × 25 × 25 in the $\Gamma$- centered sampling technique for the structural optimization^56^. The convergence condition of the ground-state is set to be 10^–8^ eV between two consecutive simulation steps, and all atoms could be fully relaxing during the geometric optimization until the Hellmann–Feynman force acting on each atom was smaller than 0.01 eV. Based on the ground-state Kohn–Sham wave functions and corresponding eigenvalues, the many-body effect is obtained within GW approximation (G_0_W_0_)^57^. In which, the cutoff energy for the response function was set to 250 eV, 8 × 8 × 8 $\Gamma$-centered kpoints sampling was used to represent reciprocal space. The wannier interpolation procedure performed in the WANNIER90 code^58^ was used to plot the quasi-band structure.

The optical properties of solid, associated with the interaction between light and the electronic excitations of a system, are described by the macroscopic dielectric function $\varepsilon \left(\omega \right)$. According to the Fermi-golden rule^59^, the single particle excitation can be depicted by the imaginary part of dielectric functions:

$${\epsilon }_{2}\left(\omega \right)=\frac{8{\pi }^{2}{e}^{2}}{{\omega }^{2}}\sum_{vc\mathbf{k}}{\left|e\langle v\mathbf{k}|\mathbf{v}|c\mathbf{k}\rangle \right|}^{2}\delta \left(\omega -\left({E}_{c\mathbf{k}}-{E}_{v\mathbf{k}}\right)\right),$$

where the first part,${\left|e\langle v\mathbf{k}|\mathbf{v}|c\mathbf{k}\rangle \right|}^{2}$ is the square of the electric dipole moment, which is responsible for the oscillation strength of the excitation peaks, and the second part, $\delta \left(\omega -\left({E}_{c\mathbf{k}}-{E}_{v\mathbf{k}}\right)\right)$, is the joined of the density of states, which associated with the available excitation transition channels. Since the incidence photon carries negligible momentum, the peaks in the absorption spectrum can be considered direct interband transition between occupied and unoccupied states without any crystal momentum transfer.

In addition to the independent particle excitations, the presence of stable excitons strongly effects on optical properties. To evaluate a more realistic two-particle picture of optical excitations, the electron–hole interactions are also taken into account. The connection of the exciton energies ${\Omega }_{S}$ and corresponding electron–hole amplitude ${\left|e.\langle 0|v|S\rangle \right|}^{2}$ of the correlated electron–hole excitations S is governed by the standard Bethe–Salpeter equation (BSE) ^60,61^,

$${\epsilon }_{2}\left(\omega \right)=\frac{8{\pi }^{2}{e}^{2}}{{\omega }^{2}}\sum_{vc\mathbf{k}}{\left|e\langle 0|\mathbf{v}|S\rangle \right|}^{2}\delta \left(\omega -{\Omega }_{S}\right),$$

where, the k-point sampling, energy cutoff, number of bands, were set to the same values as in the GW calculation. In this step, 20 lowest valence bands and 8 highest conduction bands were considered to get the optical spectrum up to 24 eV. The full chemical and physical pictures, the physics of the selective optical absorption and the effect of the robust exciton are the main study focus of the current work.

## Results and discussions

**Figure 1:**
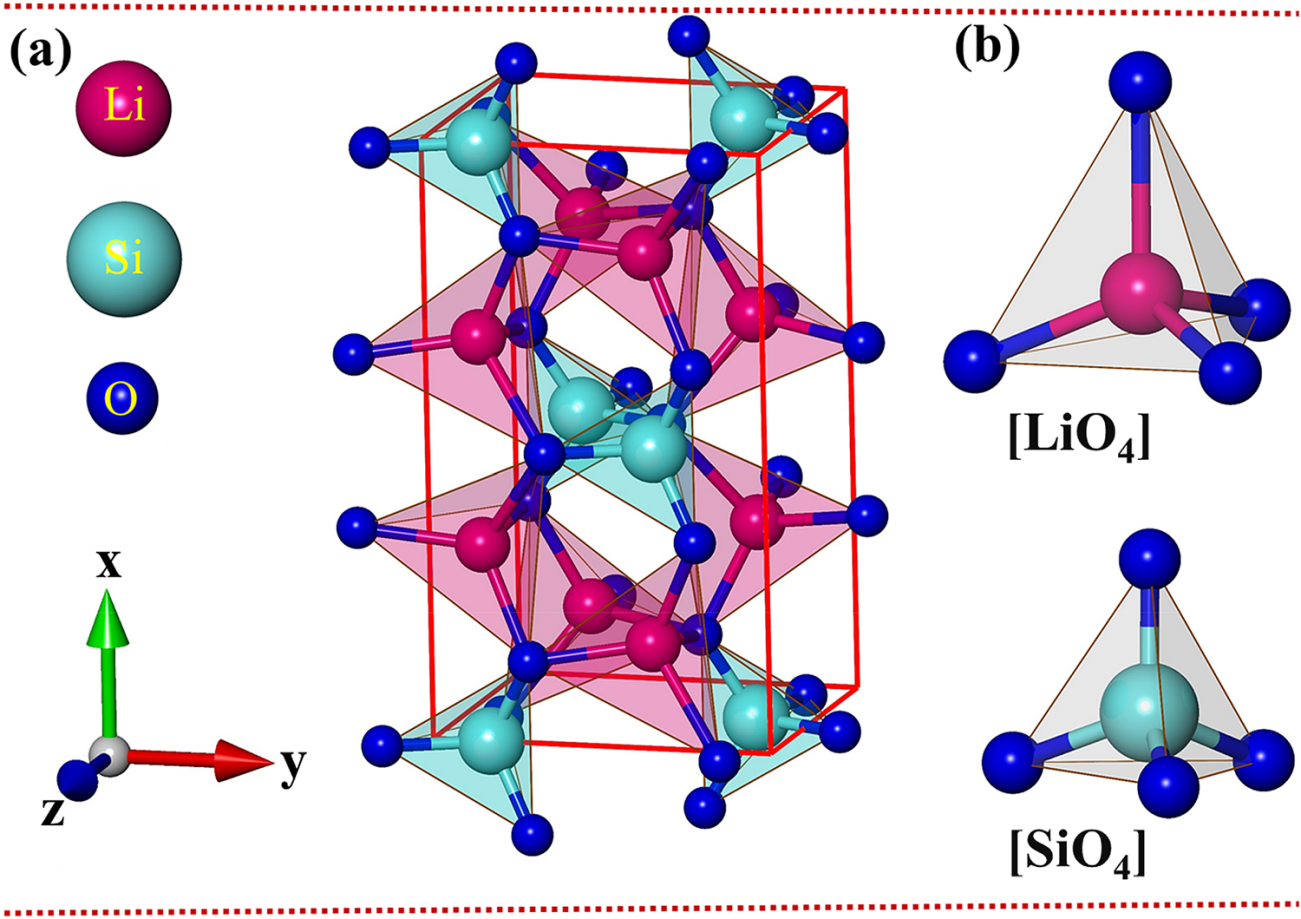
(**a**) The optimal geometric structure of the Li_2_SiO_3_ compound. Unit cell is a solid red (**b**) oxygen atoms around each Li/ Si atom.

In this work, we investigate the electronic and optical properties of the Li_2_SiO_3_ compound. This structure crystallizes in an orthorhombic structure with space group [Cmc21] (Fig. 1a). The calculated lattice constants of this compound are 9.467 Å, 5.440 Å and 4.719 Å for x-, y- and z-directions, respectively, which are close to the value in recent experimental^62^ and theoretical investigations^63^. The conventional Li_2_SiO_3_ cell contains 24 atoms (8-Li, 4- Si and 12-O atoms), where each Lithium/Silicon atom is tetrahedral coordinated and bonded to four oxygen atoms, (Fig. 1b). The basic structural unit is comprised of 32 Li–O and 16 Si–O chemical bonds, in which the bond length fluctuation ranges are ∼ 1.94 Å–2.20 Å and ∼ 1.61 Å–1.70 Å for the former and the latter, respectively. Obviously, the extreme non-uniform chemical/physical environment will be responsible for the anisotropic optical properties and evidence the complex orbital-hybridizations in the chemical bonding.

**Figure 2:**
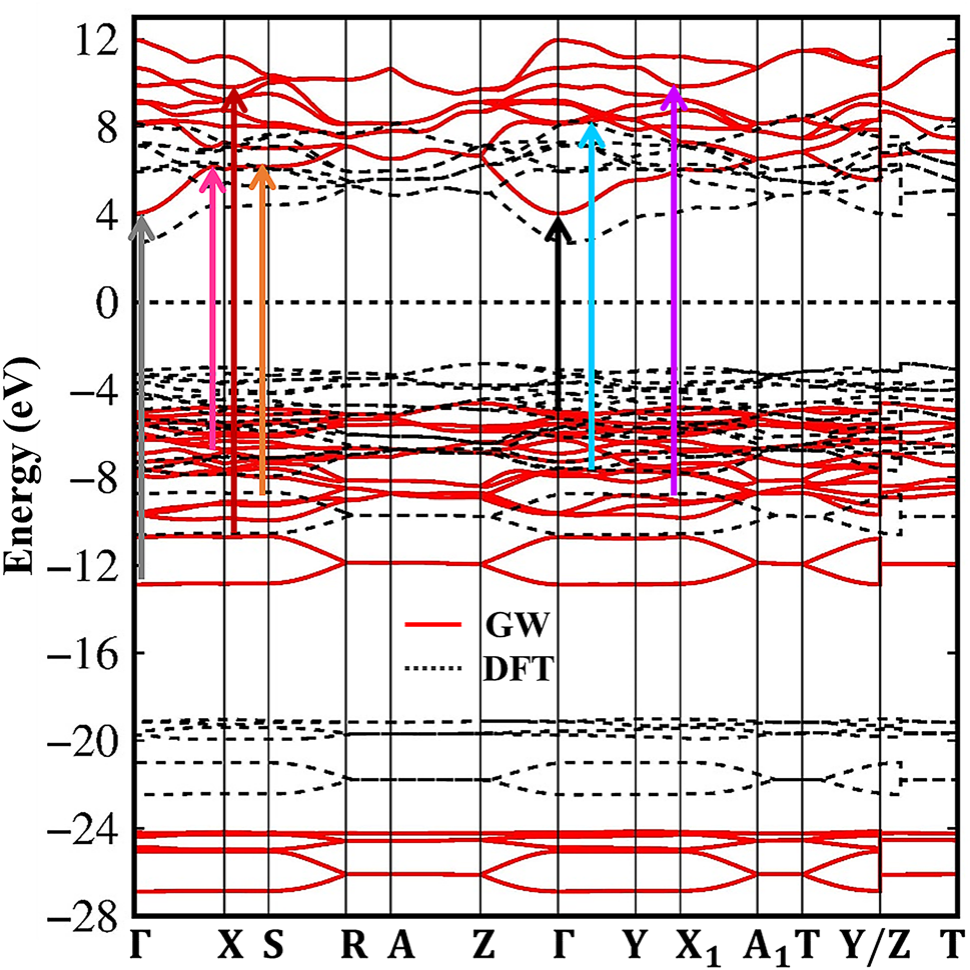
Quasi-particle band structure of the Li_2_SiO_3_ compound (the solid red line) under vertical excitations of colored arrows. The DFT band structure (the dash black line) is also added for comparisons.

The energy spectrum along high symmetry points of this material is calculated and shown in Fig. 2. As can be seen from the diagram, the Li_2_SiO_3_ compound displays rich and unique electronic properties, the high asymmetry of the valence hole and the conduction electron states is observed within a wide range of energy (− 28 eV to 13 eV). The presence of many energy-sub-bands is contributed by various atoms and orbitals. Band edge-states which are the extreme/saddle points in the energy-wave-vector space, situate at the highly symmetric points and the other wave vector between them. These critical points in the energy spectrum will create strong van Hove singularities and therefore are responsible for prominent peaks in the optical absorption spectrum. Very interestingly, the highest occupied state and the lowest unoccupied state, respectively, are located at the Z and Γ points, leading to a wide indirect gap of 8.1 eV (the solid red line in Fig. 2). This gap is significantly larger than our previous DFT calculation of 5.1 eV (the dash black line in Fig. 2), which indicates an enhancement electron–electron interaction. Obviously, the low screening leading the excitonic effects are very robust and excitonic states are well below the quasiparticle band gap.

**Figure 3:**
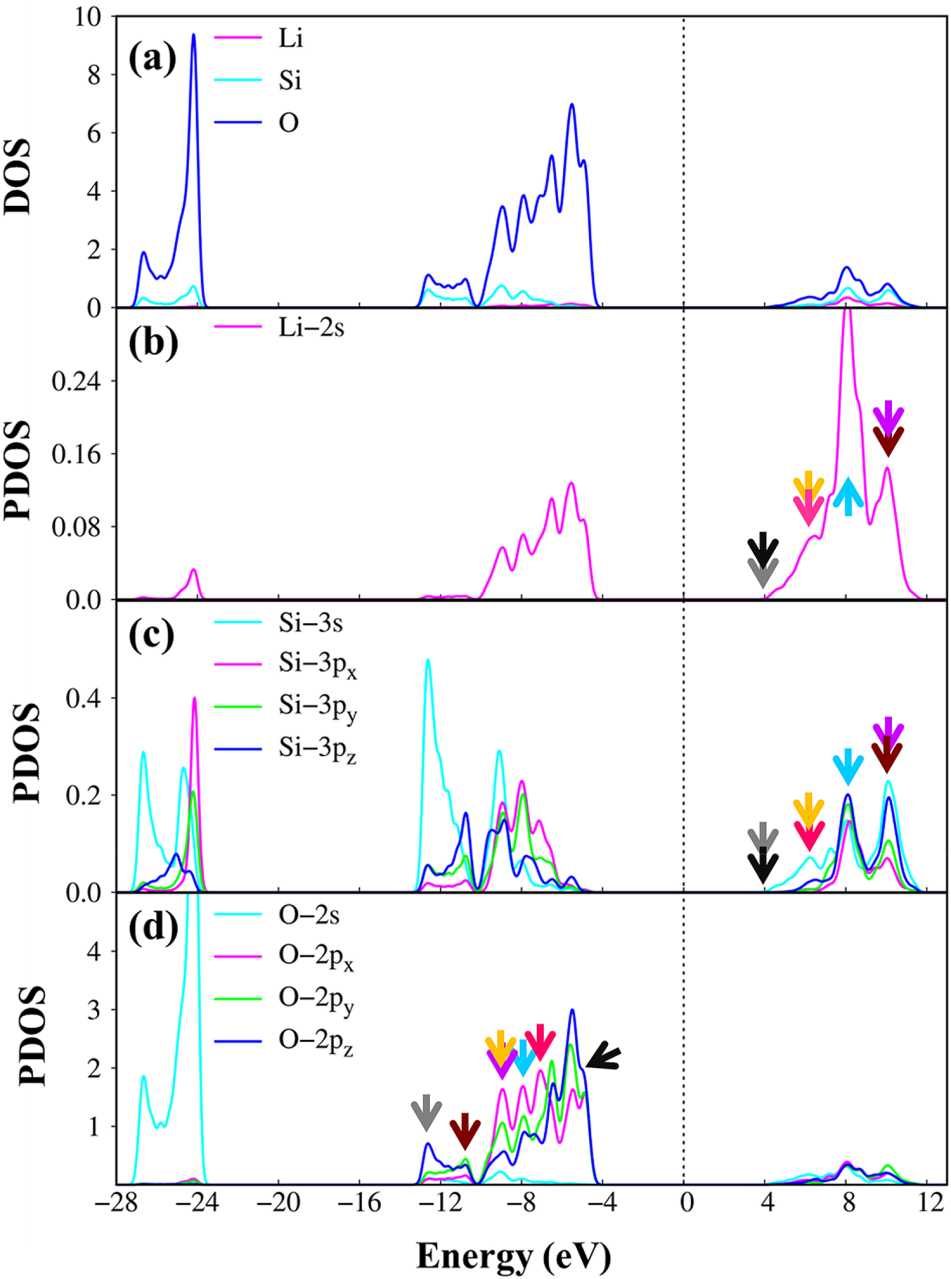
(**a**) Density of states under Li, Si, and O atoms (pink, cyan and blue curves). Projected density of states corresponding to (**b**) Li-2 s orbitals (pink curve), (**c**) Si-(3 s, 3p_*x*_, 3p_*y*_, 3p_*z*_) orbitals (cyan, pink, green and blue curves), and (**d**) O-(2 s, 2p_*x*_, 2p_*y*_, 2p_*z*_) orbitals (cyan, pink, green and blue curves).

The orbital character of the Li–O and Si–O chemical bonds, which dominate the essential electronic properties, can be sufficiently elucidated from the atom and orbital-projected density of states (DOS). The main characteristic of their special structures is governed by the energy dispersions in the energy band structure and the dimensionality of the material. As for the three-dimensional Li_2_SiO_3_ compound, the Van Hove singularities mainly come from the extreme/ saddle points and the dispersionless of energy sub-bands. This leads to the formation of the shoulder structure and pronounced asymmetric peaks (Fig. 3a). Furthermore, the considerable mixed density of states of difference atoms/orbitals (Fig. 3b–d) clearly evidences the very strong coupling of specific orbitals. Depend on the co-existence of specific atoms and orbitals, the energy spectrum of Li_2_SiO_3_ can be divided into five categories: (I) 6.5 eV < E^c^ < 13 eV is dominated of Li (2 s), O (2 s, 2p_x_, 2p_y_, 2p_z_) & Si (3 s, 3p_x_, 3p_y_, 3p_z_) orbitals, (II) 4 eV < E^c^ < 6.5 eV belong to the coupling of Li (2 s), O (2 s, 2p_x_, 2p_y_, 2p_z_) & Si (3 s) orbitals, (III) − 10 eV eV < E^v^ < − 4 eV, (IV) − 13.5 eV < E^v^ < − 10 eV is co-dominated of Li (2 s), O (2p_x_, 2p_y_, 2p_z_) & Si (3 s, 3p_x_, 3p_y_, 3p_z_) orbitals, and group (V) below − 24 eV is dominated of Li (2 s), O (2 s) & Si (3 s, 3p_x_, 3p_y_, 3p_z_) orbitals. The combination of the current results with the atom dominated-band structure and the spatial charge density before & after chemical modification (Figs. S1 and S2 in Supplementary information) can identify the presence of single-hybridization of Li (2 s)–O (2 s) orbitals and the multi-hybridization of Li (2 s)–O (2p_x_, 2p_y_, 2p_z_) & Si (3 s, 3p_x_, 3p_y,_ 3p_z_)–O (2p_x_, 2p_y_, 2p_z_) orbitals in the Li_2_SiO_3_ compound.

**Figure 4:**
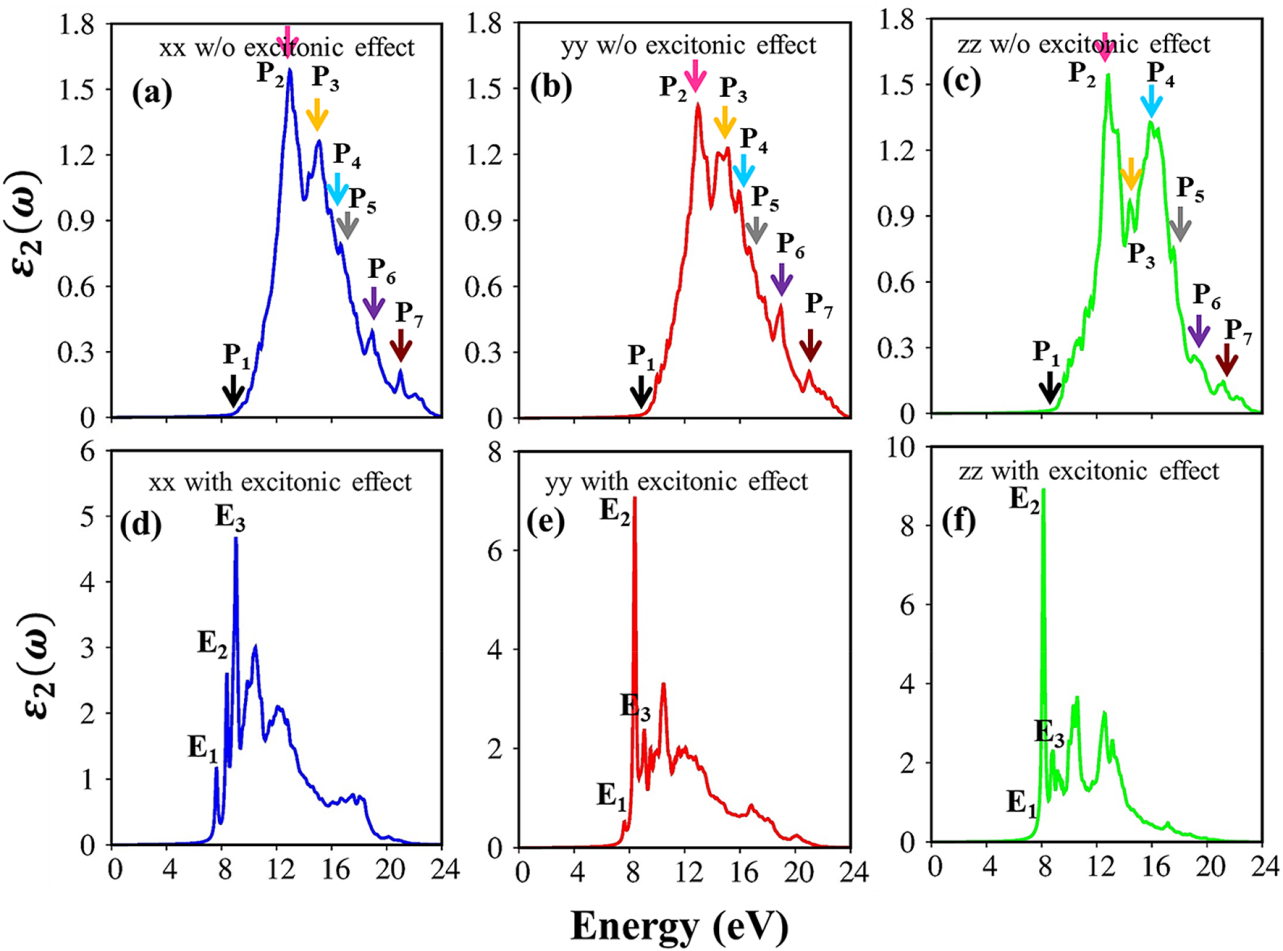
Comparison of the imaginary part of dielectric function (**a**–**c**) without (w/o) and (**d**–**f**) with excitonic effect under xx-/yy-/zz- polarization directions, respectively.

The calculated optical spectra of the Li_2_SiO_3_ compound without excitonic effects are shown in Fig. 4a–c. Because of the non-uniform physical/chemical environment in orthorhombic structure, it presents a serious change under the three different polarizations. The threshold frequency, also called the optical gap, around 8.7 eV for independent particle excitations, this value is little larger than the fundamental indirect gap ${E}_{g}^{i}$ = 8.1 eV (Fig. 2) due to the conservation of the momentum. In addition to the threshold frequency, there also exist various non-vanishing shoulders and/or pronounced peaks. The optical excitation mechanism is achieved through the state-of-the-art analysis of the concise physical and chemical pictures in the atom-dominated band structure, atom-/orbital-van Hove singularities, and strong optical responses. The arrowheads in the energy band structure (Fig. 2) describe the vertical promotion of electrons, the distinct colors of arrows in the orbital-projected density of states (Fig. 3) denote the corresponding chemical characters; the orbital hybridizations related to these optical responses are successfully identified and shown in Table 1. For example, the vertical excitation from the highest extrema to the flat energy sub-band at the vicinity of $\Gamma$-center point leading to the threshold frequency, the electron promotion from the shoulder structure in DOS/the parabolic dispersion in band structure create the shoulder structures in ${\varepsilon }_{2}\left(\omega \right),$ the transition from the strong van Hove singularities/the flat energy sub-bands will create strong prominence optical excitation peaks.

**Table 1:** Prominent absorption structures: Frequencies, colored indicators and identified orbital hybridizations with and without electron–hole interaction in Si–O bonds.

Label	Energy (eV)	Distinct colors	Specific orbital hybridizations in Si–O bonds
E_1_	7.8	Red	Si(3s)–O (2p_x_, 2p_y_, 2p_z_)
E_2_	8.2	Blue	Si(3s)–O (2p_x_, 2p_y_, 2p_z_)
E_3_	8.7	Green	Si(3s)–O (2p_x_, 2p_y_, 2p_z_)
P_1_	8.7	Black (Threshold)	Si(3s)–O (2p_x_, 2p_y_, 2p_z_)
P_2_	13	Pink	Si(3s)–O (2p_x_, 2p_y_, 2p_z_)
P_3_	15	Orange	Si (3s, 3p_x_, 3p_y_, 3p_z_)–O (2s, 2p_x_, 2p_y_, 2p_z_)
P_4_	16	Cyan	Si (3s, 3p_x_, 3p_y_, 3p_z_)–O (2p_x_, 2p_y_, 2p_z_)
P_5_	16.7	Grey	Si (3s)–O (2p_x_, 2p_y_, 2p_z_)
P_6_	19	Purple	Si (3s, 3p_x_, 3p_y_, 3p_z_)–O (2s, 2p_x_, 2p_y_, 2p_z_)
P_7_	21	Brown	Si (3s, 3p_x_, 3p_y_, 3p_z_)–O (2s, 2p_x_, 2p_y_, 2p_z_)

**Figure 5:**
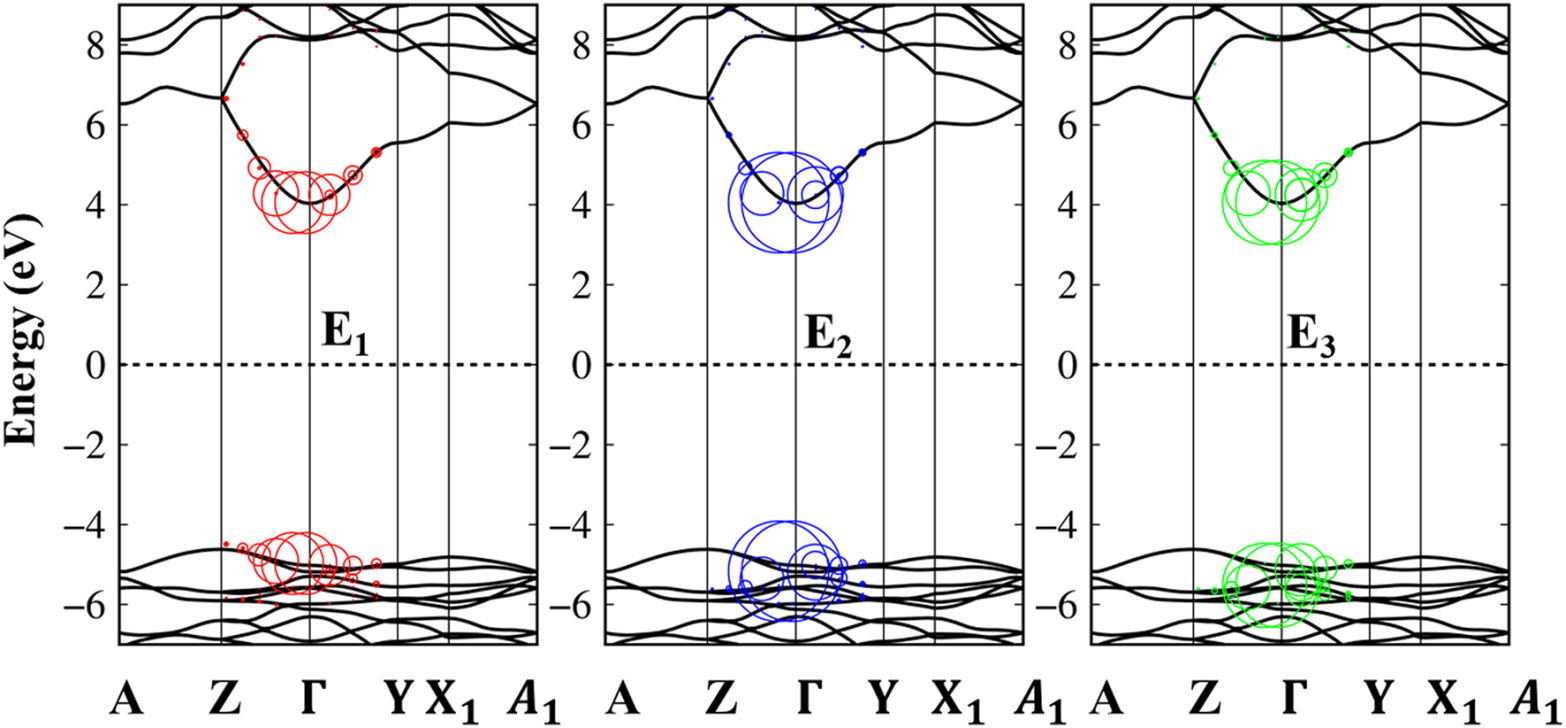
The exciton wave functions of Li_2_SiO_3_ compound are plotted as a fat-band structure. The red, blue and green circles indicate E_1,_ E_2_, E_3_ exciton peaks in the imaginary-part of dielectric functions. The black lines in the back- ground indicate the Quasi-particle band structure and the Fermi energy is set to zero at the middle of conduction and valence bands.

The bottom panels of Fig. 4 also showed the calculated spectra including excitonic effects (electron–hole interactions). The electron–hole attractions shift the oscillator strength towards lower photon energies by about 1.8/1.7/1.75 eV under x-/y-/z-directions and thus the optical threshold frequency of 6.9 eV are consistent with the experiment result^25^. The great redshift indicates very strong electron–hole interactions, and therefore, the excitonic states may stable at the room temperature. Furthermore, the presence of extra three peaks (E_1_, E_2_, E_3_) (Fig. 4d–f) below the band gap are built from the coherent superposition quasi-electron and quasi-hole pair at the extreme band edge states. To elucidate the orbital character of three-first prominence peaks, the fat band picture of exciton states indicates in Fig. 5, which covers the red, blue and green circles at E_1,_ E_2_, E_3_ peaks in the imaginary part of the dielectric function. Generally, these excitons are mainly originated from the O (2p_x_, 2p_y_, 2p_z_) to the Si (3 s) transitions (Table 1) between three last valence bands and the first conduction band. The wave vector contributing most of these excitonic peaks are mostly located at the $\Gamma$ point, where, the band edge states are parabolic curves. We do not discuss the higher excitonic peaks inside the quasi-particle band gap since they have relatively small oscillator strength and their scattering mechanism is very complicated. Based on this procedure, the orbital hybridizations associated with the specific optical transitions can be thoroughly understood. This viewpoint could be developed for other emerging materials as well.

**Figure 6:**
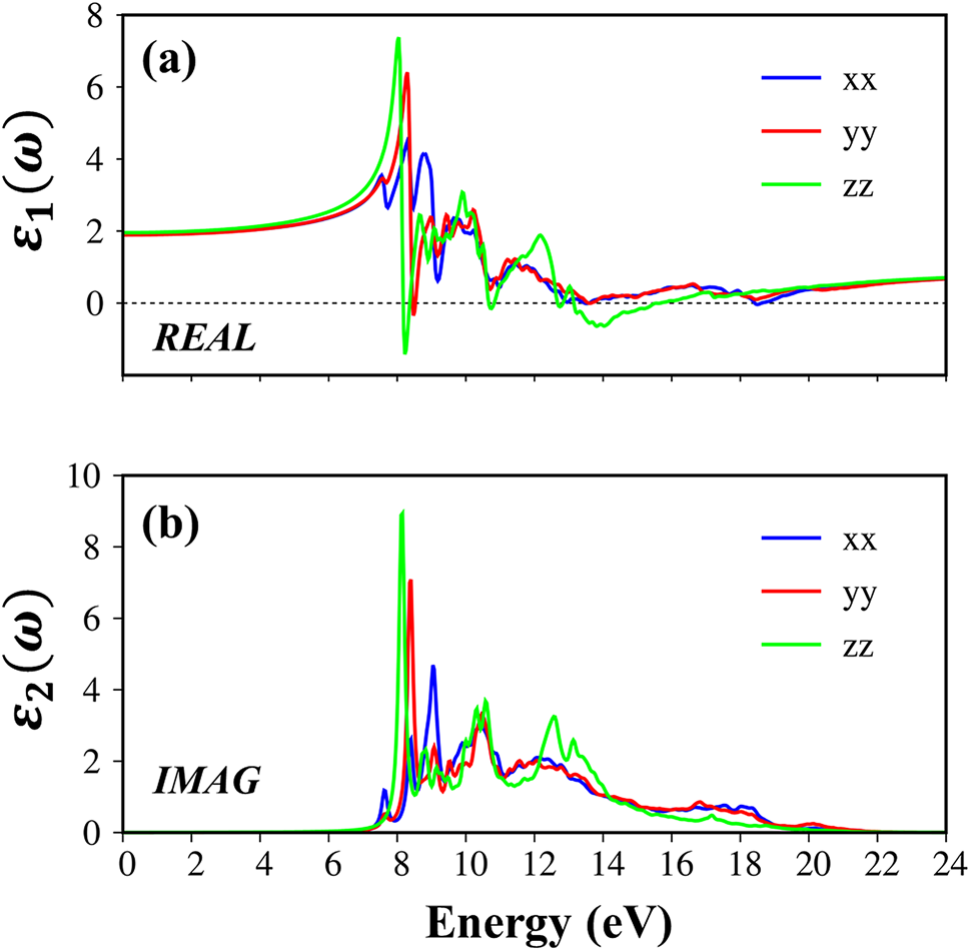
(**a**) The real-part and (**b**) imaginary-part of dielectric functions with the excitonic effects under x-/y-/z- polarization directions (the blue, red and green curves).

**Figure 7:**
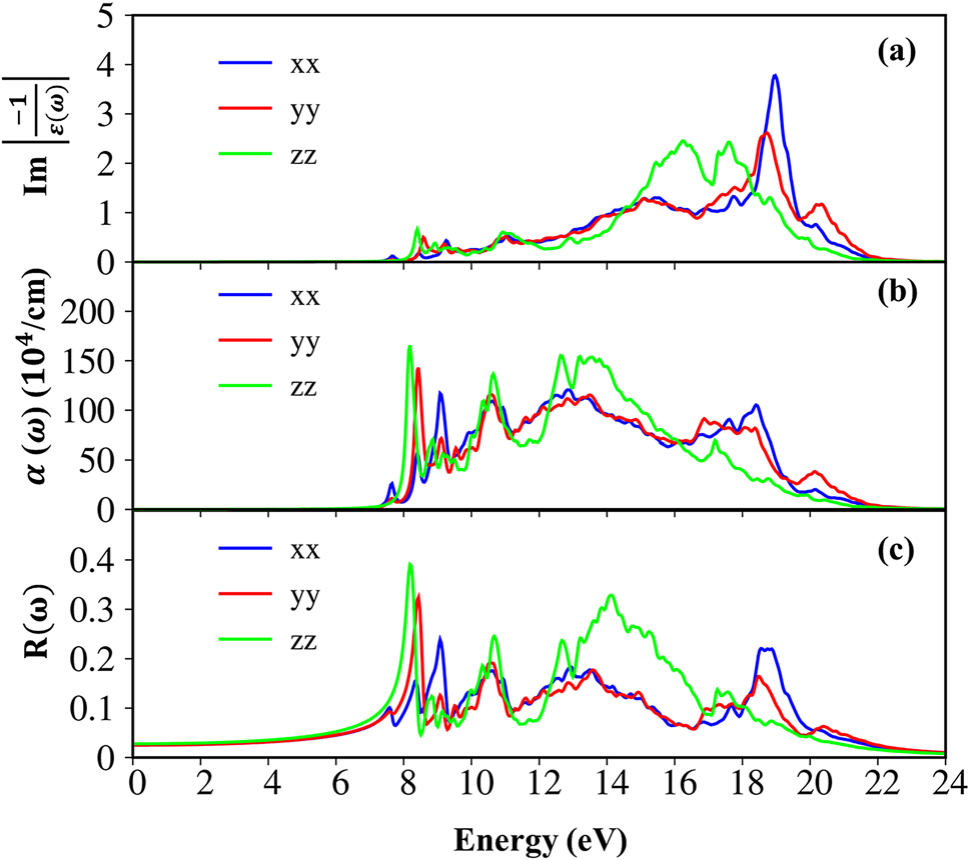
The various optical properties with excitonic effect (**a**) energy loss functions, (**b**) absorption coefficients and (**c**) reflectance spectra under the three electric-polarization directions (the blue, red and green curves).

The real part ${\varepsilon }_{1}(\omega )$ of the dielectric function relate to the imaginary part ${\varepsilon }_{2}(\omega$) through the Kramers–Kronig relationship^64^. Under this relation, the pair pronounced peaks of the real part exist simultaneously with the prominent structures of the imaginary one as present in Fig. 6b. Within the inactive region, the real part is weakly dependent on the energy. As an example, the background dielectric constants,${\varepsilon }_{1}$(0), are about 1.9–2.0 for the x-, y- and z- directions and almost stay dispersionless for frequencies less than the optical gap, through which, the low energy reflectance spectrum $R\left(\omega \right){=\left|\frac{\sqrt{{\varepsilon }_{1}\left(0\right)}-1}{\sqrt{{\varepsilon }_{1}\left(0\right)}+1}\right|}^{2}$ and the vanishing range of absorption coefficients could be identified. Contrarily, during the excitation events, the real part is very sensitive to a change of the photon energy. Very interestingly, at some frequencies, the real part almost vanishes. The simultaneous existence of the zero points of the real part with very weak single-particle transitions indicated corresponds to the strong Plasmon modes—a very small Landau damping (discussed in detail in Fig. 7a). For example, the single particle excitation at 19 eV, 18.5 eV and 17 eV for x, y and z-directions, respectively, are candidates for the resonances. On the other hand, the zero points for the z-direction at 8.1 eV combine with a very strong inter-band transition, and therefore, correspond to the serious Landau damping. Far away the active optical excitations, the real-part of dielectric function of three-directions becomes unity, the average value for x-/y-/z-directions is about 0.9. The slightly different of ${\varepsilon }_{1}(\infty )$ indicate a very interesting optical phenomena and has far-reaching research signification.

The energy loss function (ELF), being defined as Im = $\left|\frac{-1}{\varepsilon (\omega )}\right|$ ^65^ is the screened response function due to the various valence charges of Li, Si and O atoms. Each spectral peak in the ELF corresponds to the Plasmon mode, collective excitation of a certain valence charge. As we mentioned before, the zero points in the real part, ${\varepsilon }_{1}(\omega )$ (Fig. 6a) is also one way for explaining the coherent behavior of the free electrons. However, due to the combination with remarkable ${\varepsilon }_{2}(\omega$) (Fig. 6b) at certain zero points, the prominent peaks in the ELF may be better in this scenario. As clearly indicated in Fig. 7a, the strongest peak is located at frequency ${\omega }_{p}$ = 19.0/18.5/17.0 eV for the x-/y-/z-directions of electric polarizations due to almost un-damping of the Plasmon resonances. These most prominent/important peaks are contributed from the significant orbitals of Li-2 s, Si- (3 s, 3p_x_, 3p_y_, 3p_z_) and O- (2p_x_, 2p_y_, 2p_z_). Since the distributions mostly appear at very low energy below − 24 eV and hence, the contribution of the O-2 s orbital to this mode is not considered. In addition to most pronounced peaks, few Plasmon modes with relatively weak intensity exits owing to the considerable Landau damping.

The absorption coefficient and the reflectance of solid-state materials are two general optical phenomena and clearly reflect the principal characteristics of single and coherent excitations. As evident in Fig. 7b,c, when photon energy is smaller than the onset frequency, the absorption coefficient $\alpha \left(\omega \right)$ is vanishes due to lacking of the electronic excitation, while the reflectance R(ω) is weakly dependent on the frequency with the reflective index is equal 0.25 $\left(\sim {\left|\frac{\sqrt{{\varepsilon }_{1}\left(0\right)}-1}{\sqrt{{\varepsilon }_{1}\left(0\right)}+1}\right|}^{2}\right)$. However, beyond the thresh-hold frequency, both $R\left(\omega \right)$ and $\alpha \left(\omega \right)$ are changing significantly and sensitively to the excitation events. As for the absorption coefficient, the rapid increasing of these curves contributed by the various inter-band transitions, especially, the most obvious change at the Plasmon frequencies due to the participation of the collective excitations. The inverse values of the absorption coefficient are in the range of 60 Å–400 Å, indicating that the EM waves propagate in the medium are easily absorbed by the rich electronic excitations. On the other hand, the reflectance significantly enhances and displays large fluctuation. The abrupt prominence corresponding to the Plasmon mode appears at ${\omega }_{p}$. For example, the reflectance has a 22% variation under the E//xx case (the blue curve) at the resonance frequency.

The rich optical properties of the Li_2_SiO_3_ compound, such as the onset of excitonic peaks, a lot of prominent absorption structures, and the strongest coherent excitation mode at ${\omega }_{p}$ > 16.5 eV could be obtained by various optical measurements. For example, by combining the absorption, reflectance or transmission measurements with Kramers–Kronig relation, the frequency-dependent optical excitations could be verified.

## Conclusions

We performed first-principle calculations of the geometric, electronic properties and excitonic effect in optical properties of the Li_2_SiO_3_ compound. Under the state-of-the-art analysis, a concise physical/chemical picture and the crucial characteristics of this ternary substance are achieved. The Li_2_SiO_3_ compound exhibits special properties, such as the non-uniform physical/chemical environments, wide-indirect quasi-particle energy gap and atom-/orbital-projected density of states generated by the orbital hybridizations. Consequently, the distinct orbital hybridization related to the optical response can be identified. The outstanding optical properties involve the un-isotropic optical behaviors under three electronic dipole directions, the robust excitonic states with a largely reduced optical gap (${E}_{g}^{O}$= 6.9 eV) much smaller than the band gap (${E}_{g}^{i}$ = 8.1 eV), strongly modified single particle interband transitions, the low reflectance/high transparency of electromagnetic waves for $\omega$ < ${E}_{g}^{o}$, a high absorption coefficient, various prominent single-particle excitation peaks, and the pronounced peaks in ELF at the plasmon resonance frequency ${\omega }_{p},17 eV-19 eV$. The main optical features of the Li_2_SiO_3_ compound could be verified by the experimental optical spectroscopy measurements. The current theoretical framework could be developed for further studies of other emerging materials.

## Supplementary Information

Supplementary Information
