# Supplementary material for: Excitonic effects in the optical spectra of Li2SiO3 compound
Source: Sci Rep. 2021 Apr 8;11:7683. doi: 10.1038/s41598-021-87269-w (PMC8032783; doi:10.1038/s41598-021-87269-w)
Supplement: Supplementary file 1 — Supplementary Information [file 41598_2021_87269_MOESM1_ESM.docx]

**Supplementary information**

**Excitonic Effects in The Optical Spectra of Li_2_SiO_3_ Compound**

Nguyen Thi Han^1,2,*^, Vo Khuong Dien^1^, Ming-Fa Lin^1,3,**^.

*^1^Department of Physics, National Cheng Kung University, 70101 Tainan, Taiwan*

*^2^Department of Chemistry, Thai Nguyen University of Education, 20 Luong Ngoc Quyen, Quang Trung, Thai Nguyen City, Thai Nguyen Province, Vietnam*

*^3^Hierarchical Green-Energy Materials (Hi-GEM) Research Center, National Cheng Kung University, Tainan, Taiwan.*

[*^*^han.nguyen.dhsptn@gmail.com*](mailto:*han.nguyen.dhsptn@gmail.com)*, ^**^mflin@gmail.ncku.edu.tw*

**
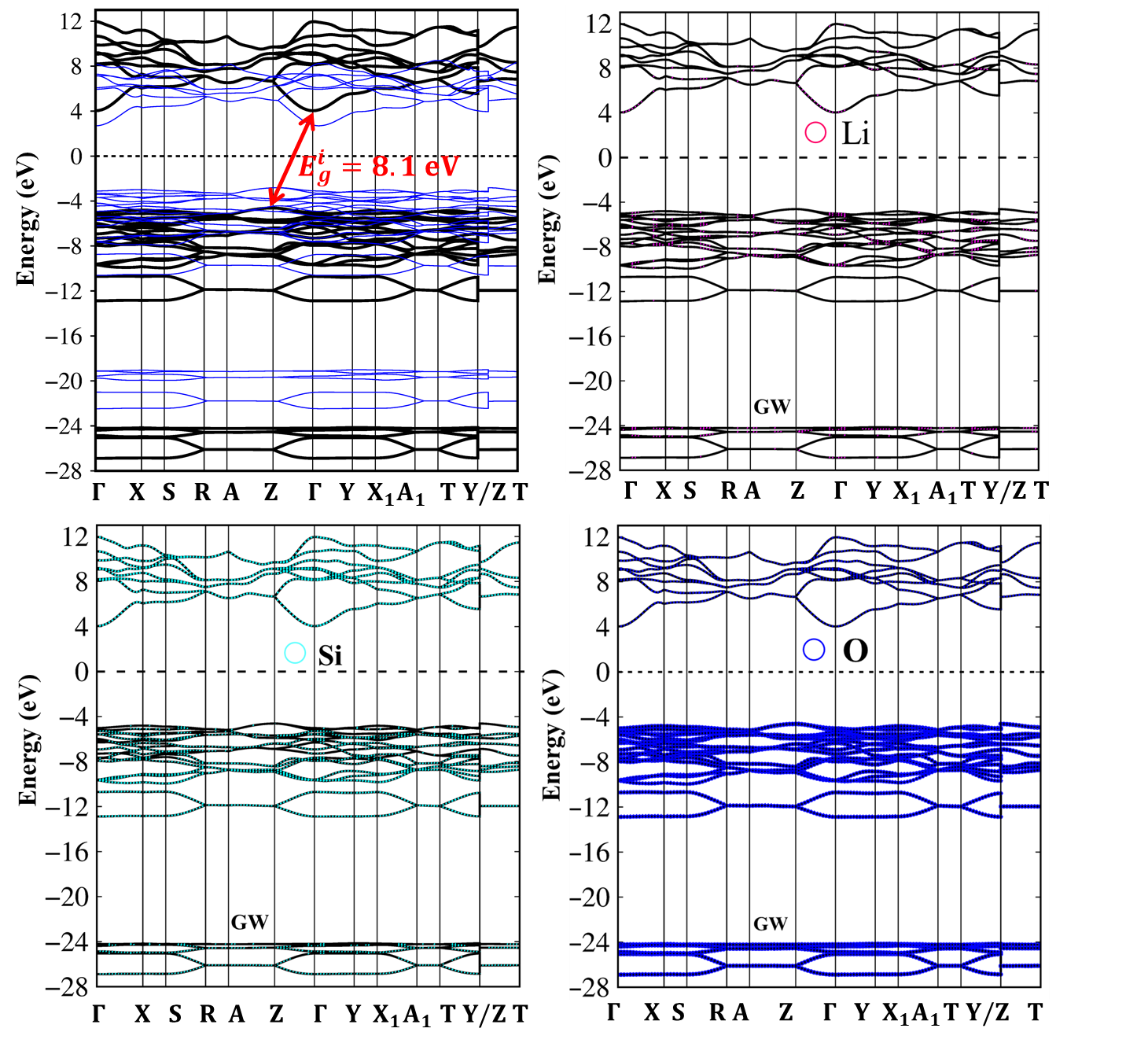
**

**Fig. S1.** (a) Comparison between quasi-particle band structure (GW) and band structure DFT along the high-symmetry points in the wave-vector space, with (b) Li-, (c) Si- and (d) O-atom dominances (pink, cyan and blue circles respectively).

In addition to the band structures, the atom dominated-energy spectrum also provides partly useful information about orbital hybridizations. For the entire band structure, the Li atoms^’^ contribution is negligible. Yet they cannot be ignored since in the absence of the Li-O chemical bonds, some noticeable features will disappear. According to the domination of the various atoms and orbital as shown in **Fig. S1(b)-(d),** the electronic band structure could be systematically divided into five sub-groups: (I) 6.5 eV < E^c^ < 13 eV, (II) 4 eV < E^c^ < 6.5 eV, (III) -10 eV < E^v^ < - 4 eV, (IV) -13.5 eV < E^v^ < -10 eV, and (V) -28 eV < E^v^ < -24 eV. Obviously, the V group below -24 eV is separated by a huge gap from the III group. Through the combination of these dominant parts the charge density distribution, the atom and orbital-projected density of states, and the fully the concise chemical/physical pictures in Li-O, Si-O chemical bonds could be identified. To date, Angle-resolved photoemission spectroscopy (ARPES) is utilized for examination of the energy dispersions and the band egde states in the occupied states. Apparently, the complicated orbit hybridizations in Li_2_SiO_3_ would create a daunting challenge in the examination of the energy sub-bands.


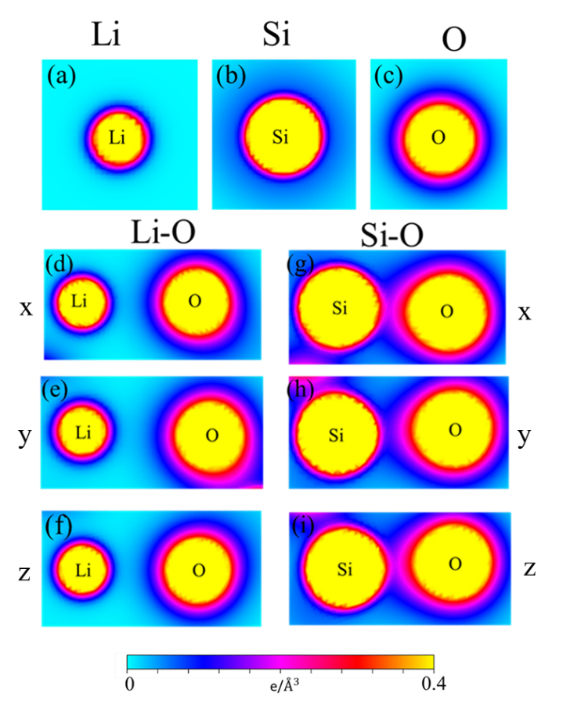


**Fig. S2**. Comparison of the isolated Li/ Si/ O atoms respectively (a)/ (b)/ (c). The charge density distributions related to the signiﬁcant orbital hybridizations in Li-O and Si-O bonds, respectively (d)/(e)/(f) and (g)/(h)/(i) along the x-/y-/z-directions for the shortest bonds.

In order to generalize the orbital hybridization between the constituents, the charge density distribution of the Li, Si and O atoms before/after modification were performed. As presented in **Fig. S2(a),** the localizations of the charge on isolated Li atom (the yellow and pink parts), correspond to the 1s and 2s orbitals. The similar but wider charge accommodation is associated with (1s, 2s) and (2p_x_, 2p_y_, 2p_z_) orbitals of the O atoms **(Fig. S2(c)).** After combination to form the chemical bonds, the inner/outer regions of the Li and O atoms of the Li-O chemical bonds along three electric-polarizations **(Figs. S2(d)-(f))** present the small but finite obvious deformations, especially for the shortest ones. This reflects the presence of non-negligible single (2s-2s) and remarkable multi 2s-(2p_x_, 2p_y_, 2p_z_) orbital interactions between these atoms. As for the Si-O chemical bonds, the highest charge density distribution residing around Si atom arises from the (1s, 2s, 2p_x_, 2p_y_, 2p_z_, 3s) and (3p_x_, 3p_y_, 3p_z_) orbitals **(Fig. S2(b))**. Similar but stronger than Li-O, the Si-O bonds are presented by the significant changes of the yellow- pink part combined with the obvious deformation of the pink region **(Figs. S2(g)-(i))** of the Si and O atoms, these propose the significant hybridization of the (3s, 3p_x_, 3p_y_, 3p_z_)-(2s, 2p_x_, 2p_y_, 2p_z_) orbitals.
